# Bacterial DNA Recognition by SERS Active Plasma-Coupled Nanogold

**DOI:** 10.1021/acs.nanolett.2c02835

**Published:** 2022-10-27

**Authors:** Vasyl Shvalya, Aswathy Vasudevan, Martina Modic, Mohammad Abutoama, Cene Skubic, Nejc Nadižar, Janez Zavašnik, Damjan Vengust, Aleksander Zidanšek, Ibrahim Abdulhalim, Damjana Rozman, Uroš Cvelbar

**Affiliations:** † Department of Gaseous Electronics (F6), Jožef Stefan Institute, Jamova cesta 39, SI-1000 Ljubljana, Slovenia; ‡ Jozef Stefan International Postgraduate School, Jamova cesta 39, SI-1000 Ljubljana, Slovenia; § Department of Electrooptics and Photonics Engineering and the Ilse Katz Center for Nanoscale Science and Technology, School of Electrical and Computer Engineering, Ben-Gurion University of the Negev, Beer Sheba 84105, Israel; ∥ Centre for Functional Genomics and Bio-Chips, Institute of Biochemistry and Molecular Genetics, Faculty of Medicine, University of Ljubljana, Zaloška 4, SI-1000 Ljubljana, Slovenia

**Keywords:** DNA genomic ratio, plasma electrochemical reduction, coupled plasmonic nanogold, DNA Raman fingerprints

## Abstract

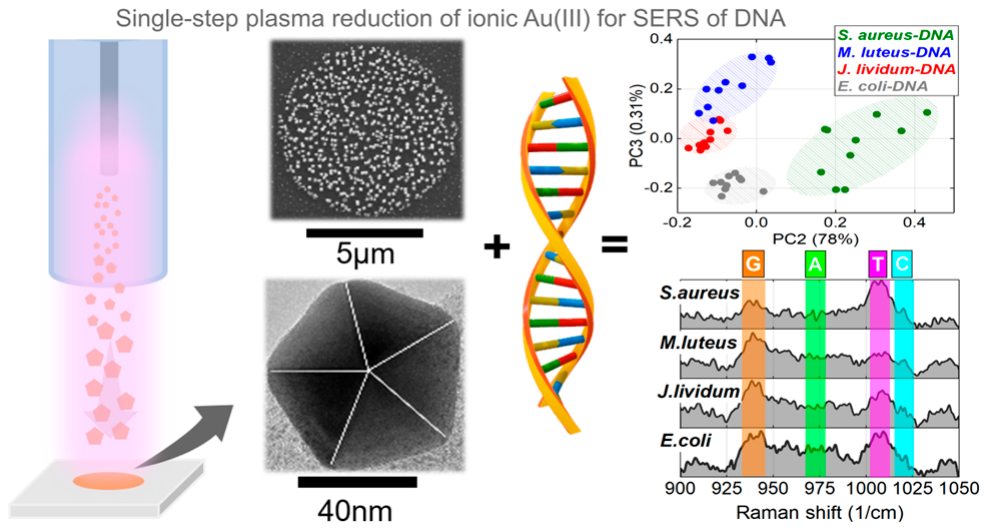

It is shown that surface-enhanced Raman spectroscopy (SERS) can identify bacteria based on their genomic DNA composition, acting as a “sample-distinguishing marker”. Successful spectral differentiation of bacterial species was accomplished with nanogold aggregates synthesized through single-step plasma reduction of the ionic gold-containing vapored precursor. A high enhancement factor (EF = 10^7^) in truncated coupled plasmonic particulates allowed SERS-probing at nanogram sample quantities. Simulations confirmed the occurrence of the strongest electric field confinement within nanometric gaps between gold dimers/chains from where the molecular fingerprints of bacterial DNA fragments gained photon scattering enhancement. The most prominent Raman modes linked to fundamental base-pair molecular vibrations were deconvoluted and used to proceed with nitrogenous base content estimation. The genomic composition (percentage of guanine-cytosine and adenine-thymine) was successfully validated by third-generation sequencing using nanopore technology, further proving that the SERS technique can be employed to swiftly specify bioentities by the discriminative principal-component statistical approach.

The rapid proliferation of pathogens^1^ defines a growing global need for the nanomedicine industry to develop analytical methods that provide in-depth investigation of biohazards at DNA level.^2,3^ It is a significant challenge to find, tune, and successfully exploit an approach that can be reliable for DNA fragments. Surface-enhanced Raman spectroscopy (SERS) has begun to be applied to the task, making the first experimental steps toward proving its practical consistency when dealing with DNA structural analysis.^4,5^ Nanoscale-sized biocompatible gold crystals of various geometries and pronounced localized surface plasmon resonance (LSPR) are the most suitable materials for this purpose due to electric field confinement at nanoscale.^6−8^ By adjusting particle planar and spatial alignment as well as excitation characteristics, photon scattering enhancement efficiency can be >10^7^, favoring single-molecule level Raman acquisition within a plasmon-active nanovolume.^9−11^

Recently, LSPR has assisted in screening binding energy of single proteins, providing evidence that SERS could play a key role in bionanosensing devices.^12^ With SERS, not only is accurate DNA detection possible,^13^ but also Raman identification of separated single nucleotides such as adenine (A), guanine (G), cytosine (C), and thymine (T).^14^ However, studies regarding DNA classification at bacteria/virus level where fragments are composed of nucleobase mixtures have not been published in the literature. Frequently, the reason for this is related to insufficient plasmonic signal enhancement generated by commercially available gold nanoparticles (AuNPs). Nanocolloids prepared by single-step approaches like laser ablation (no specificity in NP shape),^15^ or more complex methods like thermal-based electrochemical reduction^16^ (additional reducing agent involved) are not characterized by sufficient stability or adhesion on the supporting substrate. Analytical difficulties can be attributed to nonhomogeneous NP distribution over the surface, leading to problems with SERS hot-spot populations.

In this work, this problem is addressed by designing a novel, highly efficient plasmonic arrangement to accomplish the discrimination of bacterial DNA (Figure 1). Following the strategy of single-step reduction of liquid water-diluted hydrogen chloroauric acid precursor by electron donation, nanoparticle formation was accelerated significantly and more importantly, without involving any chemical reducing agents.^17^ By applying the plasma–vapor interaction technique,^18,19^ we synthesized AuNPs and controllably deposited them on a flat silicon substrate, forming unique circular clusters with abundant NP dimers and chains.

**Figure 1:**
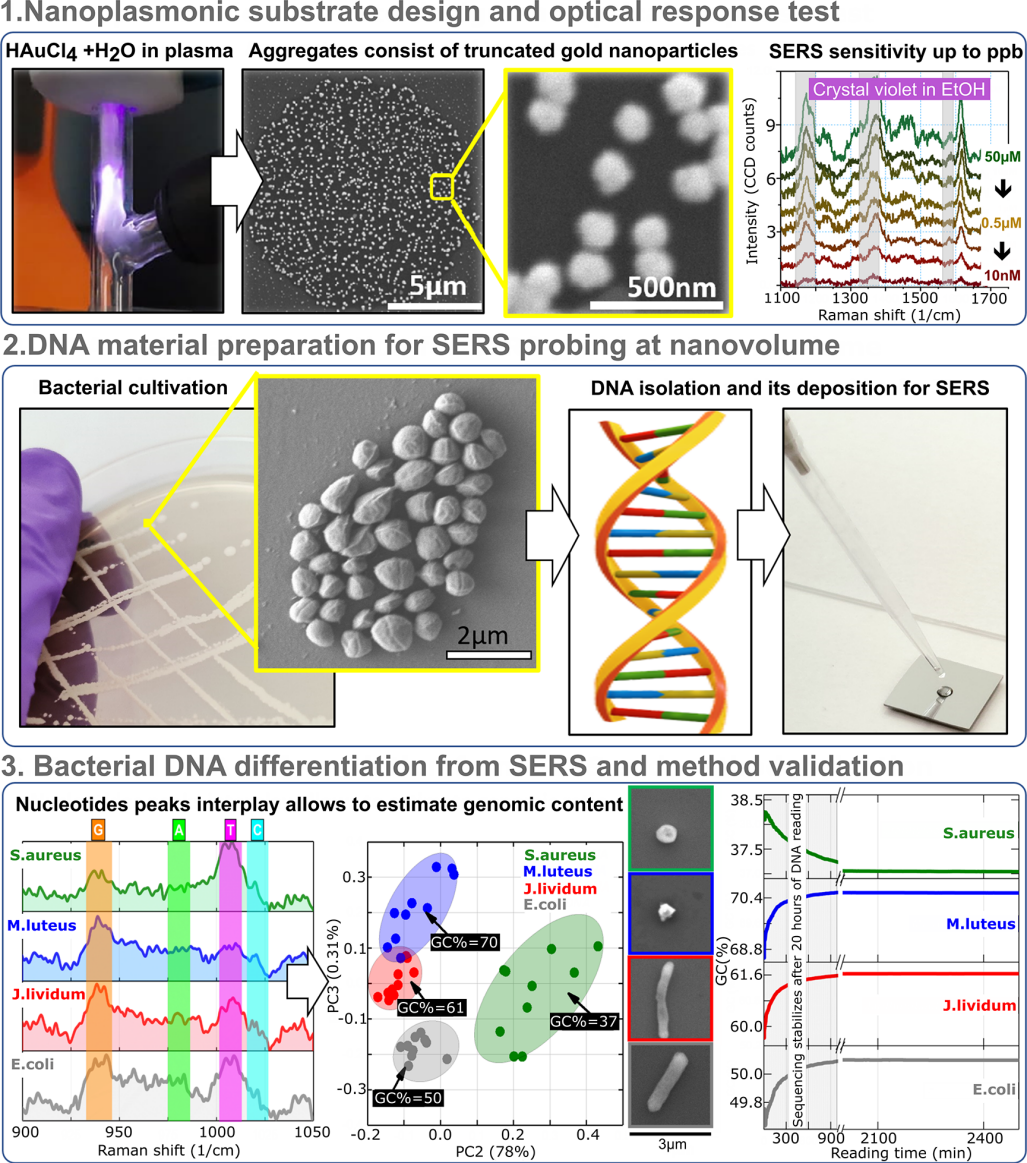
Research methodology for SERS bacterial identification. Simplified scheme of the research steps: (1) Atmospheric pressure plasma-jet system operating on Ar/He gas mixture was used for nanogold synthesis and their coupling on the silicon wafer, with a subsequent substrate sensitivity test. (2) Cultivation of bacterial strains, followed by DNA fragment isolation via extraction protocol (see Supporting Informatioin) and preparing DNA as a liquid analyte in buffer solution for SERS. (3) SERS-measured DNA sample spectra were mathematically elaborated to be processed by a principal component analysis statistical approach. The genomic ratio of GC% versus AT was estimated from peak intensities, and the numbers obtained were compared to the long-read nanopore DNA sequencing method.

As observed, the designed substrate substantially overperforms Au-coated flat silicon substrates, helping improve the SERS chip’s optical response to allow the collection of well-resolved vibrational fingerprints of DNA fragments extracted from *Escherichia coli, Janthinobacterium lividum*, *Micrococcus luteus*, and *Staphylococcus aureus* bacteria. Utilizing a spectral range, where four nucleotide bases reveal prominent separated Raman peaks and take a fundamental genomic ratio guanine-cytosine (GC%) as a key spectral difference, all DNA bacterial strains were successfully SERS-distinguished by the principal component analysis (PCA) method. GC contents were validated by genome sequencing nanopore technology to further validate the SERS method as a potential approach to specify bioentities reliably by DNA vibrational features and statistical PCA processing.

## Gold Nanoaggregates Designed by Single-Step Plasma-Assisted Reduction

**Figure 2:**
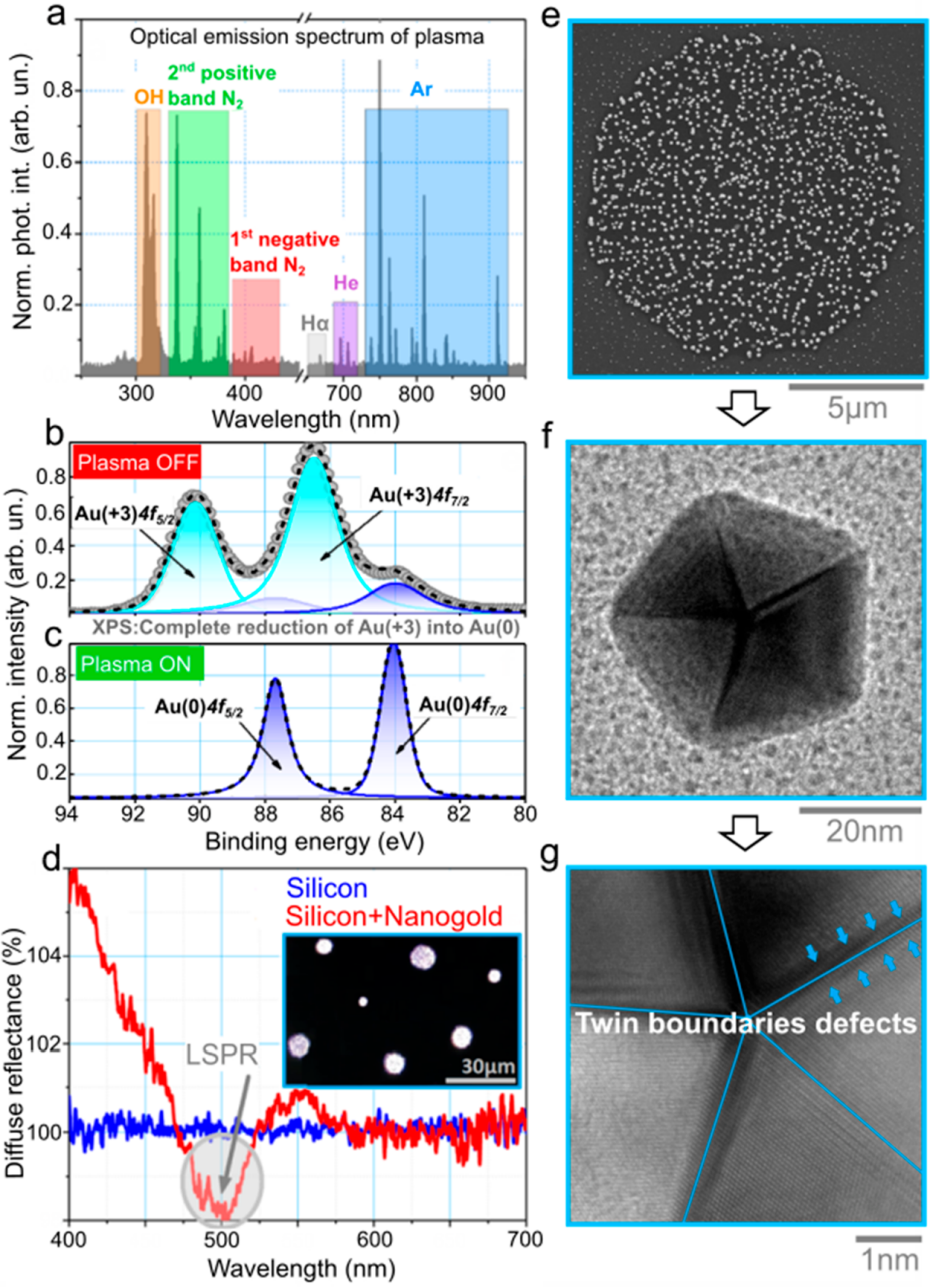
Synthesis of truncated nanogold aggregates. (a) Optical emission spectrum of the ignited plasma. (b) X-ray photoelectron spectroscopy (XPS) spectrum of the dried raw precursor, revealing corresponding peaks for gold with ionic Au(III) (cyan areas) and metallic Au(0) (blue areas) core level contributions. (c) Au peak deconvolution obtained from XPS data after plasma-jet deposition. No ionic shares are obtained. (d) Diffuse optical reflectance of the flat silicon taken as a reference (blue curve) and silicon with deposited nanogold (red curve). The inset is an optical image of gold clusters over flat silicon taken in a dark-field configuration using a halogen bulb as a light source. (e) Backscattered electron-scanning electron microscopy (BE-SEM) micrographs of Au particles on top of the silicon wafer obtained after atmospheric pressure plasma jet treatment of the water-diluted hydrogen chloroauric acid precursor. (f,g) Transmission electron microscopy (TEM) micrograph pair, revealing truncated NP shape with indicated twin boundary structural defects.

Plasma accelerates chemical reactions, skipping intermediate oxidation to allow nearly instant transformation of ionic gold Au(III) into its neutral state Au(0).^17^ In the current situation, reduction occurs due to electron donation inside the discharge of plasma, where hydrated (e _aq_) and free (e_gas_) electrons, together with H and OH reactive radicals, act as an electrochemical reducing factor.^20,21^ Cold atmospheric plasma electrons (<0.1 eV) are efficient in NP reduction, nucleation, and growth.^22^ Optical emission measurements collected during plasma discharge (Figure 2a) indicate the occurrence of reactive species, revealing a strong contribution of argon and helium excited lines (blue and violet boxes in Figure 2a). The obtained set of peaks placed between 320–460 nm are features linked to nitrogen lines appearing from the interaction of plasma with environmentally present nitrogen molecules. Highly reactive OH^–^ radicals found at 310 nm appear as a result of interaction between water diluted raw precursor, ambient air, and ignited plasma. These active hydrogen-free radicals and hydroxide species recombine within the plasma discharge, creating highly oxidative H_2_O_2_ species and an excess of free electrons, transforming the ionic metal with oxidation state Au(III) down to metallic state Au(0), which initiates nanocrystal growth from gold nuclei (Figure S1).

Consequently, micron-sized aggregates consisting of coupled gold nanoparticulates are obtained (Figure 2e). We suggest that each circular Au aggregation is the end-product of precursor droplet reduction, and the following hypothesis explains the process involved. The aqueous solution of HAuCl_4_ × H_2_O possesses low volatility, and its droplets are of relatively large volume. Once the solution’s volatility is increased by adding ethanol with 1:1 volume ratio to water, as demonstrated by Hong et al., then smaller particles are obtained^17^ The estimated gas temperature of 610 ± 20 K, might initiate faster evaporation of the carrier solvent, leading to faster NP growth in comparison to other reports.^22^ As observed, larger/heavier metal particles are clustered tightly within a circular area. Their mean diameter varies in the range 10 ± 4 μm (Figure S2a). It was found that an average cluster’s population is approximately 40 ± 5 within an area of about 0.1 mm^2^. The rest of the Si is decorated homogeneously by Au particles of a smaller size (<100 nm). (Figure S2b,c). A Wulff crystal shape (Figure 2f,g), e.g., “ideal truncated bipyramid”, is obtained when the equilibrium crystal is formed according to the Gibbs thermodynamic principle by minimizing the total surface free energy associated with the crystal–medium interface. Additionally, AuNPs are associated with structural defects, more precisely ∑3-type tilt twin boundaries, which will produce, based on the newly constructed morphology, dominant {111} crystal faces and will result in formation of 5-fold dodecahedral crystals.

The liquid precursor’s reduction efficiency by plasma processing is quite high, as the amount of chlorine detected by energy-dispersive X-ray spectroscopy (EDS) is nearly negligible (Figure S3a). No other contaminants are detected. Exact elemental composition numbers are collected in Table S1. No trace of chlorine on Si after plasma deposition is obtained from X-ray photoelectron spectroscopy (XPS) data either (Figure S3b). The opposite occurs for dried raw precursor material; a typical chlorine peak (Cl 2p) centered at about 199 eV is distinctly observed (Figure S3b). Deconvoluted high-resolution Au 4f XPS peaks exhibit a dominant contribution of ionic Au(III) in dried precursor, while after reduction, nanoparticles reveal a metallic state of gold Au(0) (Figure 2b,c).

## Plasma-Made Nanogold Is Highly SERS-Active

**Figure 3:**
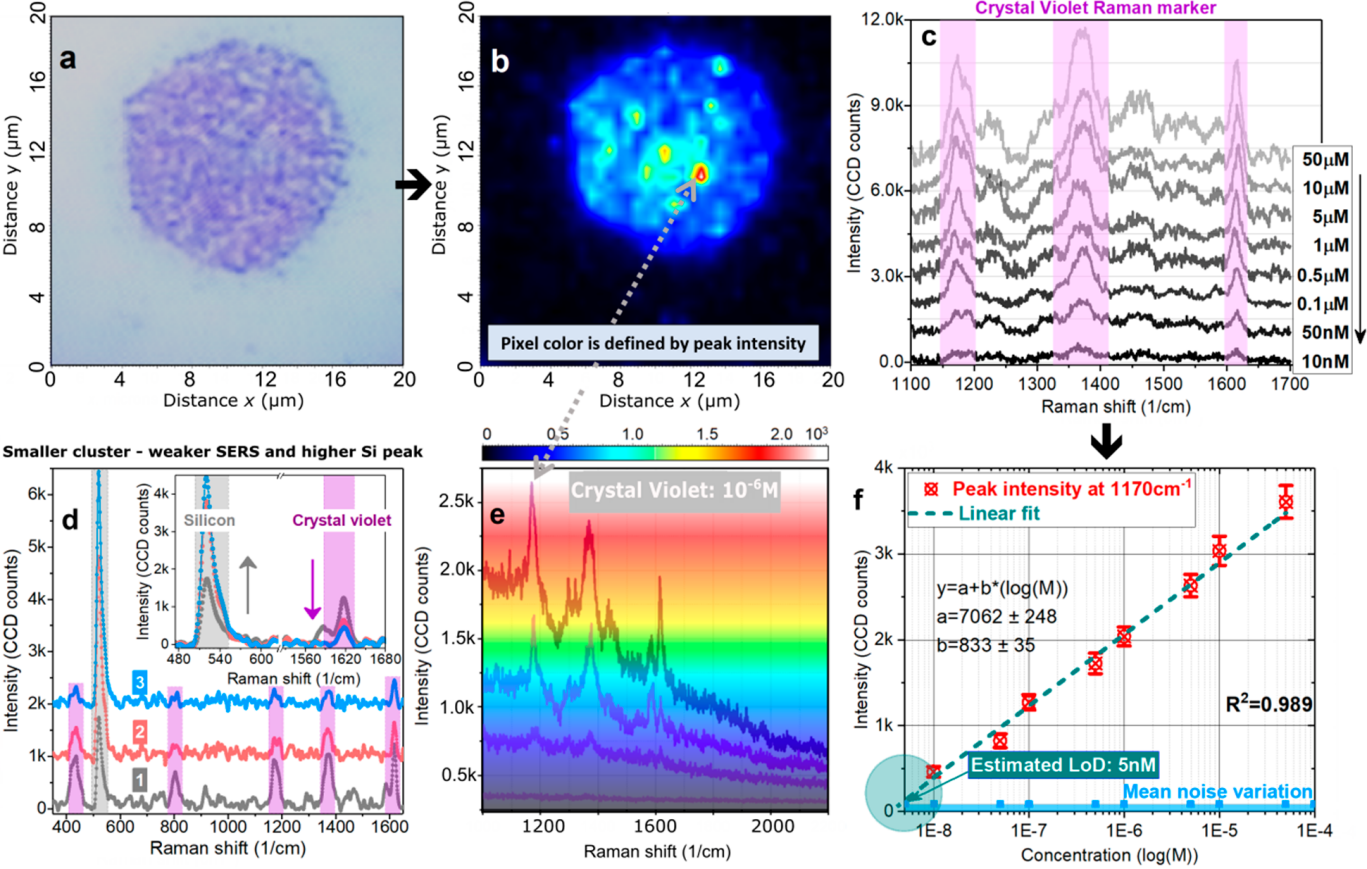
SERS optimization. (a) Optical image of an Au nanocluster on Si wafer. (b) Raman mapping of crystal violet (CV) deposited on gold decorated silicon. (c) Raman spectra of CV aqueous solution in respect to the concentrations. (d) SERS of 1 ppm of CV from different nanoclusters labeled as “1” = large, “2” = medium, and “3” = small. (e) Vibrational spectra of CV, where the intensity of a mode located at 1180 cm^–1^ defines a pixel color in (b). (f) Raman intensity of the antisymmetric stretching (CC_center_C) band at different concentrations and its linear extrapolation.

Following a reported procedure,^23^ the surface plasmon resonance was investigated by diffuse reflectance. An LSPR-driven spectrum is obtained with a wide peak 500 nm (absorption) and a shoulder rising at 550 nm (extinction) (Figure 2d). Afterward, crystal violet (CV; 10^–6^ M) was deposited (Figure 3a), and Raman mapping was performed with a total number of 400 spectra acquired (Figure 3a,b). The color of each pixel corresponds to the intensity of the characteristic antisymmetric stretching ν_as_(CC_center_C) molecular mode located at 1180 cm^–1^; shown by a colored scale (Figure 3b,e). The other two main peaks arise from the symmetric stretching vibration of the (C–C)_ring_ at 1625 cm^–1^ and the bending motion of the δ(CH)/δ_s_(CH_3_)/δ(CCC)_ring_ band at 1391 cm^–1^.^24,25^ A contour of the enhanced signal coincides precisely with the cluster’s shape, indicating its enhanced optical response (Figure 3a,b). Within the plasmonic area, signal intensity is locally increased by a few orders of magnitude. Although a higher Raman signal could be, to a certain extent, related to concertation variation, it is far more likely a consequence of increased efficiency arising from enhanced plasmon coupling. This SERS response within nanometric gaps is well understood and has been recorded numerous times.^26−28^

The analysis of microclusters of different sizes provided insight into the cumulative effect of Raman signal enhancement. Three types of circular aggregates were considered according to their outer contour radii: large, medium, and small, with an approximate diameter of 14, 8, and 2.5 μm, respectively (Figure S4). By counting all the nano-objects (ImageJ software^30^) within a single cluster, the filling factor *f* slightly increases with area expansion according to the following trend *f* = 0.10 → 0.12 → 0.14. However, *f* never goes higher than 0.2.

Next, a portion of Au dimers and chains were estimated (Figure S4a1–c1, a2–c2). Regardless of the type of cluster, the amount of such agglomerates is about 30–40%. Thus, it could be believed that the bigger cluster would provide higher cumulative signal enhancement due to having a larger number of hot spots. These three clusters are SERS-compared by using a CV molecular marker (Figure 3d). The first thing to notice is how the Raman peak associated with crystalline Si (521 cm^–1^) increases with Au cluster diameter shrinkage. This is expected because the laser spot covers a larger surface (10 μm) area than just with silicon. This peak is particularly intense for *small* nanogold clusters with diameter of 2.6 μm (Figure 3d inset).

In contrast, scattering intensity corresponding to the model dye CV rises when the cluster area becomes larger. Taking a CV peak located at 1620 cm^–1^ as a reference and considering an optical response of a large cluster as 100%, the medium cluster reveals 1.9× and small Au aggregates 2.7× intensity reduction. A set of different ethanol-diluted dye concentrations were SERS-probed for a limit of detection (LoD) study. The Raman intensity of the most prominent vibrations (Figure 3c) decreases with lower concentrations. Spectra are, however, still well-resolved at minimum concentrations equal to 10 nM. A semilogarithmic linear trend of signal intensity versus solution concentration (Figure 3f) allows LoD estimation of about 5 nM. The enhancement factor (EF) is evaluated and its value jumps up to ∼10^7^, demonstrating the exceptional sensing capabilities of the substrate.

## Ultrahigh Field Enhancement Favors Accurate SERS Recording of DNA

**Figure 4:**
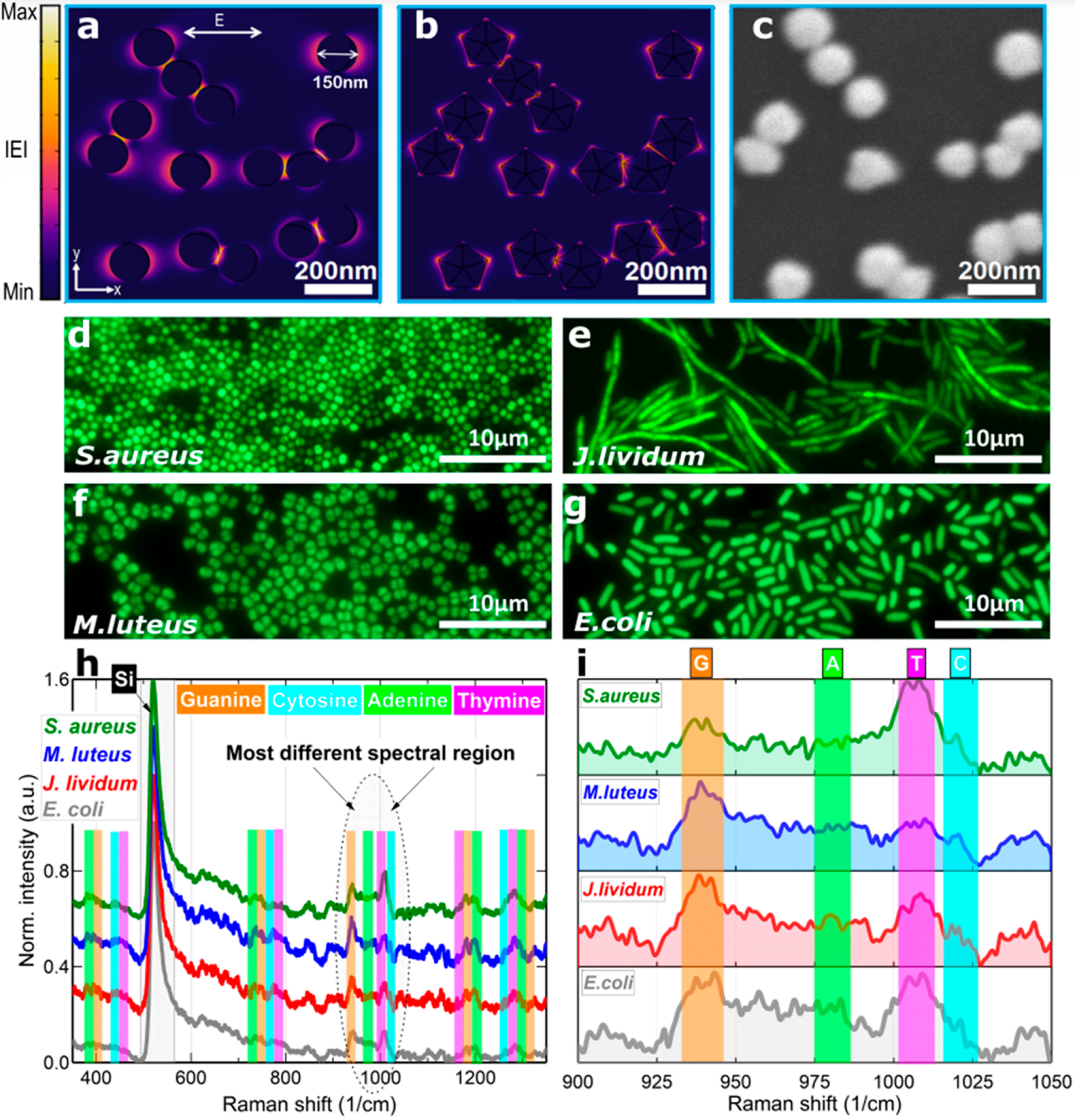
Electric field confinement ensures reliable bacterial DNA sensing. (a) Field distribution (FD) simulations inside the unit cell of the considered periodic structure with spherical particles. (b) The same FD simulation for distorted five-edged-like bipyramids. Remarks: λ = 633 nm, normal incidence and transverse electric polarization were used to simulate the gold NPs on a silicon substrate. Air was assumed to exist between the NPs and the superstrate material. For a more quantitative estimation of the field, see Supporting Informatioin. (c) SEM image of an AuNP cluster selected for real-case scenario Maxwell EM field modeling. (d–g) Microscope images of cultivated bacterial species. (h) SERS spectra of extracted bacterial DNA in the fingerprint region. (i) Enlarged part of the long-range spectra highlighting the most prominent vibrational differences in the genome content-sensitive region.

Maxwell field distribution simulations (COMSOL Multiphysics software) (Figure 4a,b) were performed using a real-case scenario of NP arrangements, where single Au nano-objects of about 150 nm in size and their geometrical interplay with dimers and chains is displayed (Figure 4c). Because particles are neither spherical nor ideal truncated bipyramids, it was decided to elaborate on two extreme boundary cases: ideal spherical and five-edged bipyramid geometries (Figure S5). Thus, a real scenario could naturally be expected to fall between the modeled results. The important evidence coming from Figure 4a is that the largest field enhancement exists within the nanometric gaps between the NPs forming the dimers and chains. Much smaller enhancement is observed around single nano-objects, supporting the significance of close-packing NPs. The influence of the distorted NP geometry accompanied by sharp edges (Figure 4b) also favors field enhancement. The field intensity (Figure 4a,b) is greater at least by a factor of ∼2 in the case of bipyramids compared to spheres due to the stronger field confinement effect near the tips (Figure S6–7).

Next, DNA fragments were extracted from four bacterial species (Supporting Informatioin) from a pair of spherically shaped (*S. aureus*, *M. luteus*) and rod-like looking bacteria (*J. lividum*, *E. coli*) (Figure 4d–g), and their genomic fragments were SERS-measured (Figure 4h,i). A sign of the successfully deposited analyte on the sensing surface can be confirmed by interference lines observed from a bright-field optical image and scattering lines in dark-field mode (Figure S8). Ten vibrational spectra for each macromolecular DNA complex were accumulated and elaborated mathematically, applying baseline subtraction, smoothing function, and data normalization (Figure S9).

Within Figure 4h, the strongest peak placed at 521 cm^–1^ is assigned to the Si substrate. Despite being quite similar in shape at first look, the spectra reveal noticeable dissimilarities across the measured range. Following the research conducted by Otto et al.,^29^ the Raman peaks of the main nucleotide bases (G, C, T, and A) were marked according to their locations. Typically, a mixture of different nucleotides is associated with plenty of vibrational modes, frequently overlapping each other within the fingerprint region. However, two spectral intervals, namely 350–450 cm^–1^ and 875–1075 cm^–1^, might be used to indicate the presence of all four nucleotides. In the low-frequency range (350–450 cm^–1^), below 400 cm^–1^, spectra are dominated by the ring bending vibrations of the structurally larger complexes consisting of two aromatic rings (G and A). Similar vibrational features of the nucleotide’s bases possessing one structural ring (C and T) are shifted toward higher frequencies and located just above 400 cm^–1^.

## Characteristic Genomic Content Is a Key Factor for Distinguishing DNA

**Figure 5:**
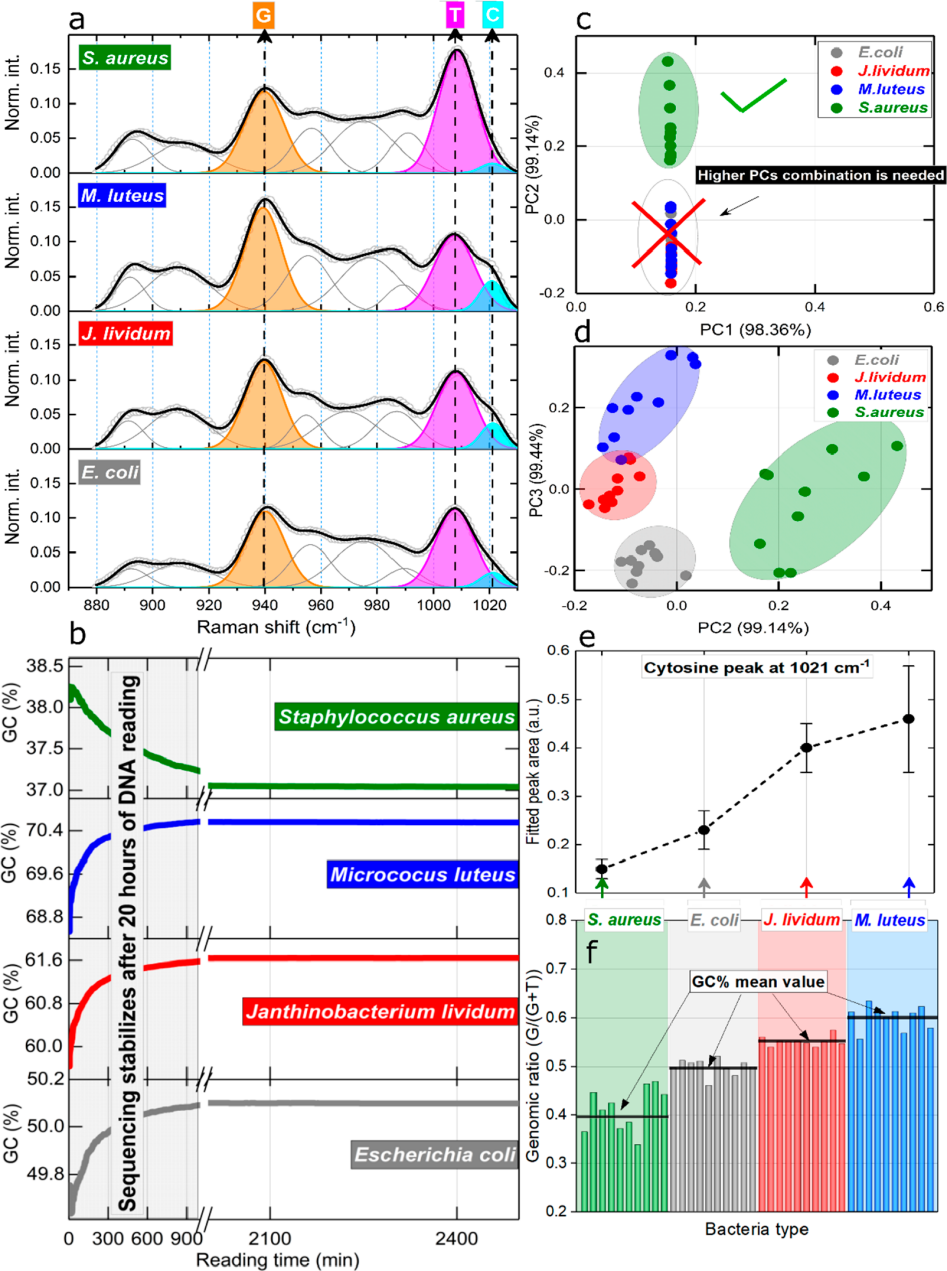
Distinguishing DNA by genomic content and data verification by DNA sequencing method. (a) SERS data with Gaussian fittings of the nucleotide modes in a specified spectral interval. (b) Sequencing technique with *in situ* GC nucleotide content during DNA nanopore reading of each bacterial DNA. (c,d) SERS PCA with clustered spectral data. (e) The fitted area of the SERS-measured C peak at 1021 cm^–1^. (f) Calculated genomic ratio of bacterial DNA using peak intensity SERS data with indicated mean values of a certain DNA type.

More distinct information is hidden in the spectral region 875–1075 cm^–1^ (Figure 4i). Here, peaks are sharper, more intense, and well separated. Referring to research by Otto et al.,^29^ the prominent Raman mode at 940 cm^–1^ originates from G structure and the peak located near 1008 cm^–1^ is attributed to T nucleic base. An evident shouldering of the T mode due to a peak at 1021 cm^–1^ is caused by a combined contribution of NH_2_^rocking^ + C_6_H^bending^ vibrations in C molecular structure of bacterial DNA.^29^ Raman features of A cannot be resolved clearly in this range (between G and T horns) due to a more complex mutual interaction with other structural mode vibrations of similar intensity, full-width at half-maximum (fwhm), and area (Figure 5a). The above-mentioned findings, especially the G and T related Raman peak intensity ratio interplay, were taken as key spectral features to run a PCA. The PCA tends to classify the spectra by creating separated data clusters with common features reflected in a loading plot (Figure 5c,d). From these two graphs, a general remark arises: PC1 vs PC2 combination, which is typically sufficient for separating two groups of samples, in our case, is not applicable to classify four different sets of bacterial DNA. A combination of higher principal components is required for adequate sample distinguishing. The graph Figure 5d composed of “PC2 (abscise)” vs “PC3 (ordinate)” contains four colored data clouds, each including ten spectra (one dot–one spectrum) correlated to a particular bacterial DNA sample. A detailed description of dimensionality reduction data processing with related figures (Figure S10–12) is in the Supporting Informatioin.

The most reasonable question to ask here is is there any relation between a fundamental genomic ratio GC% (G, C) of bacterial DNA and the Raman intensities of certain peaks within this range? To better understand the PCA results, these three characteristic peaks were analyzed regarding their intensity. After Gaussian deconvolution (Figure 5a), it can be said that in the case of *E. coli*, the Raman intensities of T and G modes are of similar height. The relevant fitting information (area, fwhm, position) of characteristic SERS modes elaborated in Figure 5a are listed in Tables S2–S4. For *M. luteus*, the G peak is much larger than T, while the opposite occurs for *S. aureus* DNA. In the case of *J. lividum*, the T mode is somewhat smaller the G vibrational peak.

The observed peak intensity behavior correlates with DNA GC% content of measured bacterial species. From fundamental biology, it is known that G always pairs with C, and T is structurally connected to A, and the experimental genomic ratio might be estimated using a relation (G/G+T), focusing on peak intensities. The (G/G+T) contents are calculated (Figure 5f), and their mean values (black horizontal line) increase in accordance with GC% data from the literature. The average GC% value for bacteria tested fall within the established ranges of *M. luteus* = 69 ± 4%, *J. lividum* = 61 ± 4%, *E. coli* = 50 ± 6%, and *S. aureus* = 39 ± 4% (Supporting Informatioin). However, the bacterial genome GC content may vary substantially by the number of cell divisions; thus, whole genome sequencing of bacterial strains was conducted as well, applying the long reads offered by Oxford Nanopore technology (Figure 5b and Table 1).

**Table 1:** A General Comparison between Nanopore Sequencing and SERS Technologies in Terms of Bioanalysis

	methods	
crucial metrics	sequencing	SERS
sensitivity (amount of sample used)	200 ng	100 ng
steps before data collection	1 – Bacteria cultivation	1 – Bacteria cultivation
	2 – DNA extraction	2 – DNA extraction
	3 – Purification of DNA	3 – Spectra recording
	4 – Preparation of libraries	4 – PCA analysis
	5 – Genome reading	
	6 – DNA fragments assembling	
	7 – Bioinformatics analysis	
selectivity	Very high	High
analysis time	Hours – Days	Seconds – Minutes
overall analysis cost	High	Low
scale and area of application	Molecular level:	Molecular level:
	DNA/RNA	DNA/RNA
	Other molecules:	Other molecules:
	--------Not applicable---------	lipids, proteins, sugars - YES
	Cellular level	Cellular level
	--------Not applicable---------	Bacteria, viruses, fungi – YES
type of data	DNA composition sensitive – YES	DNA composition sensitive – YES
	DNA topology sensitive – NO	DNA topology sensitive – YES

At the beginning of the DNA reading, error was at its largest, reaching up to 5%, but after approximately 20 h of nonstop sequencing, a stable, precise, saturated value was obtained and the final numbers for genomic content are given: *M. luteus* = 70 ± 2%, *J. lividum* = 61 ± 2%, *E. coli* = 50 ± 2%, and *S. aureus* = 37 ± 2%. The nucleotide contents obtained from SERS measurements are as follows: *M. luteus* = 59 ± 5%, *J. lividum* = 54 ± 4%, *E. coli* = 50 ± 3%, and *S. aureus* = 40 ± 6%. Interestingly, a contribution of the shouldering C peak at 1021 cm^–1^ follows the logic of the (G/G+T) ratio as well as GC%: its area/intensity is the smallest for *S. aureus* and the largest is found for *M. luteus* bacterial DNA (Figure 5e, Table 1).

A larger uncertainty in SERS results might be related to (i) smaller sample quantity compared to the amount requested for sequencing and (ii) worse sample purity of the examined sample in SERS analysis (an additional purification step is required for nanopore technology to prevent blocking of protein pores). It can be suggested that the nano-Raman technique possesses strong analytical potential for compositional/structural investigations of biological materials at molecular and cellular levels Despite pointing to a proof-of-concept, there is still room to improve the method in the presented challenge, such as reducing error and further validation of DNA SERS markers.

In summary, SERS-active micron-scale aggregates composed of truncated nanogold particulates, obtained by a plasma–driven instant reduction mechanism, can provide SERS measurements of DNA at nanograms sample quantities (100 ± 10 ng/μL). Involving no external reducing agent, plasma processing accelerated the formation of multiple nanogold dimer and chain particulates. Verified by field distribution modeling, the plasmonic substrate achieved an enhancement factor as high as ∼10^7^. Further, it allowed the specification of vibrational fingerprints of DNA fragments extracted from common bacterial species by multivariate statistical PCA analysis, linking the results to genomic content ratio. SERS is highly sensitive to the DNA environment and offers a faster, more facile, complementary pathway that enables primary data on GC% to be obtained, distinguishing DNA with smaller sample quantities, and the removal of an additional DNA purification step required in the nanopore DNA sequencing technique.

## Supporting Information Available

Description of atmospheric plasma setups and AuNPs synthesis; additional SEM images, XPS data, electric field distribution modeling, bacterial DNA extraction, principal component analysis processing, and methodology of Oxford Nanopore Technologies for DNA fragments sequencing (PDF)

## ABBREVIATIONS

DNA: deoxyribonucleic acid
SERS: surface-enhanced Raman spectroscopy
XPS: X-ray photoelectron spectroscopy
EDS: energy-dispersive X-ray spectroscopy
SEM: scanning electron microscopy
NPs: nano particles
LSPR: localized surface plasmon resonance
CV: crystal violet
FE: field enhancement
EM: electromagnetic
FD: field distribution
PCS: principal component analysis
G: guanine
T: thymine
A: adenine
C: cytosine
TM: transverse magnetic
TE: transverse electric
