# Supplementary material for: Bacterial DNA Recognition by SERS Active Plasma-Coupled Nanogold
Source: Nano Lett. 2022 Oct 27;22(23):9757–65. doi: 10.1021/acs.nanolett.2c02835 (PMC9756328; doi:10.1021/acs.nanolett.2c02835)
Supplement: Supplementary file 1 — nl2c02835_si_001.pdf [file nl2c02835_si_001.pdf]

Bacterial DNA Recognition by SERS Active Plasma-Coupled Nanogold

Vasyl Shvalya[a], Aswathy Vasudevan[a,b], Martina Modic[a], Mohammad Abutoama[c], Cene Skubic[d], Nejc Nadižar[d], Janez Zavašnik[a], Damjan Vengust[a], Aleksander Zidanšek[a,b], Ibrahim Abdulhalim[c], Damjana Rozman[d] and Uroš Cvelbar[a,b]*

[a] *Department of Gaseous Electronics (F6), Jožef Stefan Institute, Jamova cesta 39, SI-1000 Ljubljana, Slovenia, EU.*

[b] *Jozef Stefan International Postgraduate School, Jamova cesta 39, SI-1000 Ljubljana, Slovenia, EU.*

[c] *Department of Electrooptics and Photonics Engineering and the Ilse Katz Center for Nanoscale Science and Technology, School of Electrical and Computer Engineering, Ben-Gurion University of the Negev, Beer Sheba 84105, Israel.*

[d] *Centre for Functional Genomics and Bio-Chips, Institute of Biochemistry and Molecular Genetics, Faculty of Medicine, University of Ljubljana, Zaloška 4. SI-1000 Ljubljana, Slovenia*

* Email: uros.cvelbar@ijs.si

Supporting Information

Synthesis of nano-gold. Crystalline tetrachloroaurate (III) hydrate ($HAuCl_4 \times H_2O$) (99.9% metal basis, Au 49% min) was used as the raw precursor purchased from Alfa Aesar by Thermo Fisher (Kandel) GmbH, Germany. The material was weighed at the amount of 0.01 g and dissolved in 10 ml of distilled water to make the feed solution, which was heated at 60 °C for 30 min using magnetic stirring. The solution was kept aside to cool down to room temperature. After, 4 ml of the preheated solution was transferred to the nebuliser in tube contact with the atmospheric pressure plasma jet system. The experimental setup comprised two vertically aligned quartz tubes of inner diameter 3 mm and 7 mm, where the larger one was an actual plasma chamber. The smaller tube was also of a shorter length. A gold-coated copper wire was inserted, whose free tip acted as a plasma ignition point. The main inlet gas was helium flowed at a rate of 290 sccm. The He plasma was ignited at a power of 25 W operated at a frequency of 21.2 kHz. A heavier argon carrier agent was connected to a nebuliser containing a liquid precursor solution $HAuCl_4 \times H_2O$. Gas Ar with a much higher flow rate set as 1000 sccm fetched vaped precursor microdroplets, bringing them into contact with the generated plasma. Note that no grounding was applied to the Si wafer. The substrate of approximate area 1 cm^2 was fixed on a moving stage motor operated by WinPC-NC software. Uniform distribution over the substrate was achieved by moving the sample stage in a zig-zag manner for 30 min. The nebuliser was constantly supplying the precursor at the rate of 1000 ml/min.

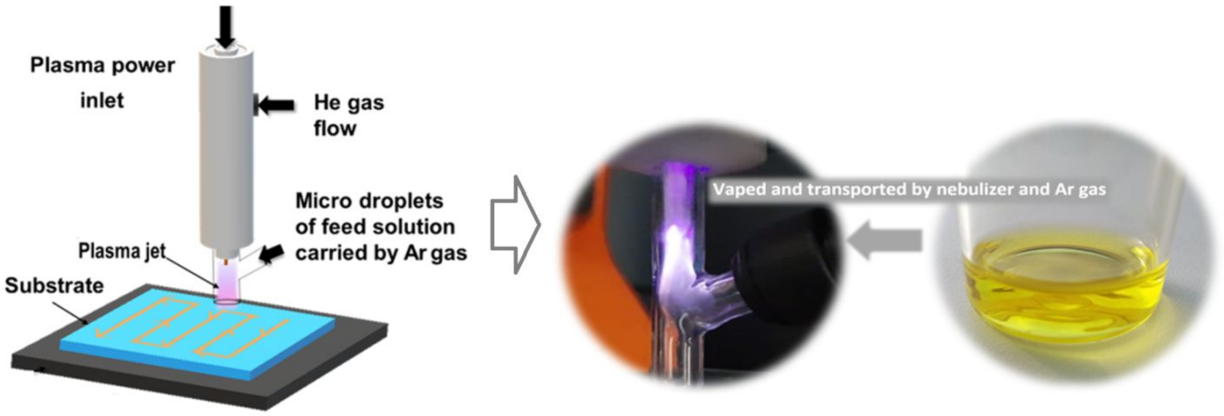

Figure S1. Plasma deposition setup schematics accompanied with photos of operando reduction–deposition process and feeding solution of water-diluted $HAuCl_4$ precursor.

SEM, TEM and size distribution analysis. Size, shape and arrangement of freshly deposited nanoparticles were investigated by exploiting a Prisma E scanning electron microscope (Thermo Fisher Scientific Inc.) in different magnifications using secondary emitted electrons accelerated at a voltage of 10 keV. Transmission electron microscopy (TEM, JEM-2100, Jeol Inc.) functioning at 200 kV was employed to characterise the gold nanoparticle's morphology. The images were collected by a slow-scan CCD camera (Orius SC1000, Gatan). Statistical data regarding the spatial distribution of nano-gold within a cluster was provided via software *ImageJ* using different circularity filters for each discrete task.[30]

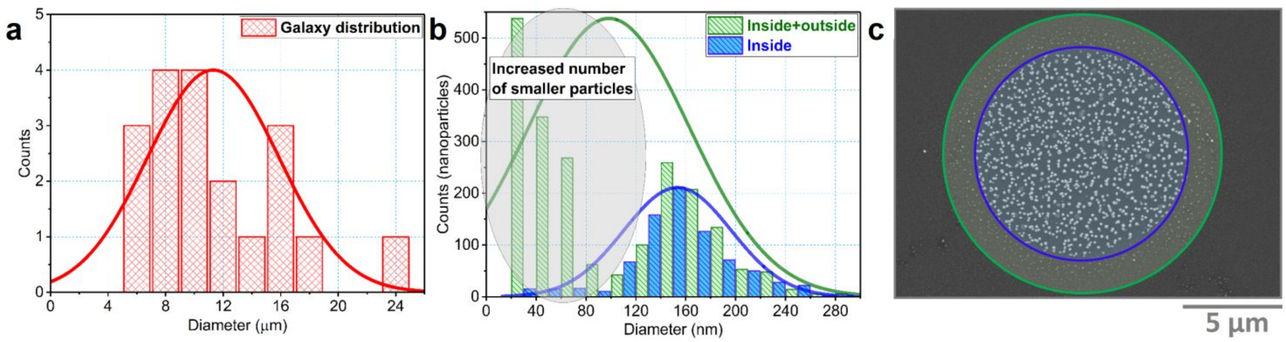

Figure S2. (a) **-** Mean size distribution of Au clusters. (b) Size distribution analysis of NPs enclosed within selected areas displayed in (c)

EDS and diffuse optical reflectance. The elemental composition of a selected area of the sample was carried out by energy-dispersive x-ray spectroscopy under acceleration voltage equal to 10 keV and an acquisition time of 120 s. A double beam UV–Vis spectrophotometer fabricated by Perkin Lambda (model 1050) equipped with a 150 mm integrating white sphere module was utilised to investigate the diffuse reflectance covering a spectral interval of 400–700 nm with a resolution of 0.2 nm.

Table S1. Surface composition of the plasma reduced gold nanoparticles on Si from EDS test.

**Element**	**Atomic %**	**Error**	**Weight %**	**Error**
Si	98.3	0.2	89.6	0.2
Cl	0.1	3.7	0.1	3.7
Au	1.6	3.7	10.3	3.7

X-ray photoelectron spectroscopy. The oxidation state of gold nanoparticles was investigated using PHI-TFA-XPS spectrometer (Physical Electronics Inc.) armed with an Al-monochromatic photon source operating at an energy of 1486.6 eV. XPS peak intensities were fitted via the OriginLab software package, using a Gauss–Lorentz function with Shirley-type background subtraction. The analysed surface area was approximately 400 μm^2.

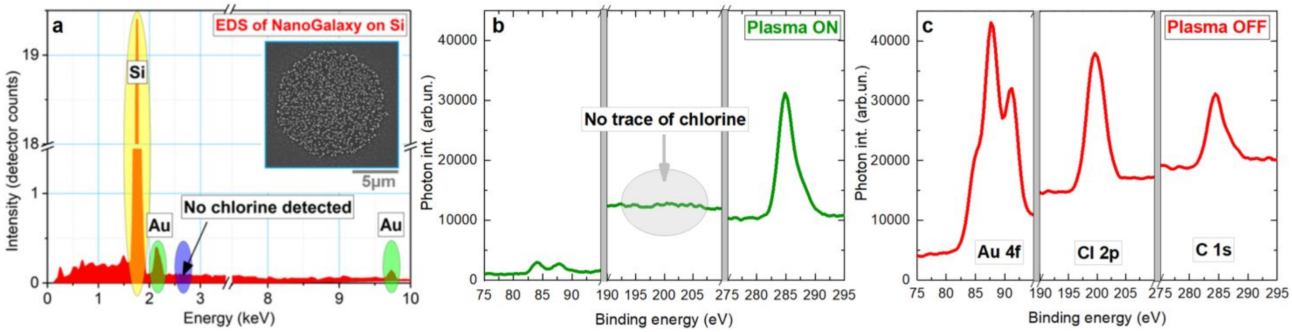

Figure S3. (a) Energy-dispersive X-ray spectroscopy (EDS) of Au-decorated silicon given in the inset. (b-c) XPS survey cut-off regions represent the absence of chlorine (Cl 2p) regarding clearly detected Au4f peaks after plasma deposition. Carbon C 1s peak is included as a reference peak.

Remarks: The curve "Plasma OFF" was recorded from a dried droplet of precursor with a focused X-ray beam on it; thus XPS signal is quite pronounced. An important piece of information here is the relative intensity of the Cl to Au peak. In the case of "plasma OFF", regime peak intensities are more or less similar, while for "Plasma ON" mode, there is no Cl 2p peak detected at all.

SERS measurements of crystal violet and DNA fragment. Vibrational spectra of model CV dye dissolved in water at different concentrations (5×10^{-5}–1×10^{-8} M) were collected using a confocal µ-Raman spectrometer (NT-MDT, model NTegra Spectra II) operating at 633 nm wavelength of a He-Ne laser. A 20× objective lens with a numerical aperture of 0.40 was used for laser beam control. At first, 1 µl of CV solution was pipetted on the sample surface and dried at ambient conditions. Then, Raman spectra of DNA samples were taken after 5 s laser exposure and an accumulation number of 3.

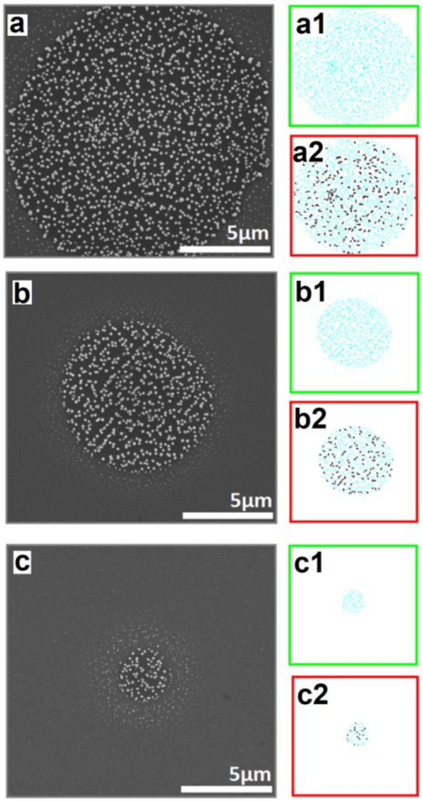

Figure S4 (a–c) BE-SEM images of gold clusters with different diameter. (a1–c1) Counted nanoparticles using ImageJ software when a "circularity" filter is in soft mode. (a2–c2) The same clusters processed with a rigorous "circularity" threshold (black particles are omitted).

Electromagnetic simulations. The distances between the different nano-aggregates are on the order of 30 μm and, therefore, no interaction is expected to occur between the individual Au clusters at visible wavelengths. Our interest is in calculating the FD within the selected area shown in **Figure 3a**. Due to the large number of NPs forming the circular pattern, simulating the FD within the micron-scale cluster would require huge computation time and resources. Therefore, two cases were considered and compared in the simulations to test the reliability of the results. First, periodic structure (along x- and y-directions) was considered in **Figure 3** from the manuscript, where the unit cells represent the part of the nano-gold aggregate which was selected to build, as much as possible, a reliable EM simulation. In other words, the unit cell must include

single NPs, dimers and NP chains to give a realistic insight into the signal enhancement origin. The modelled structure was created in AutoCAD software. Since the Au particles deposited on the silicon substrate are neither completely spherical nor bipyramidal-like shaped, it was decided to elaborate on two extreme types of NP geometries, namely ideal spheres and bipyramids of the same size (**Figure S5**). Following this modelling logic, the real case scenario will for sure fall within results obtained for extreme geometries, increasing the credibility of theory–experiment interplay.

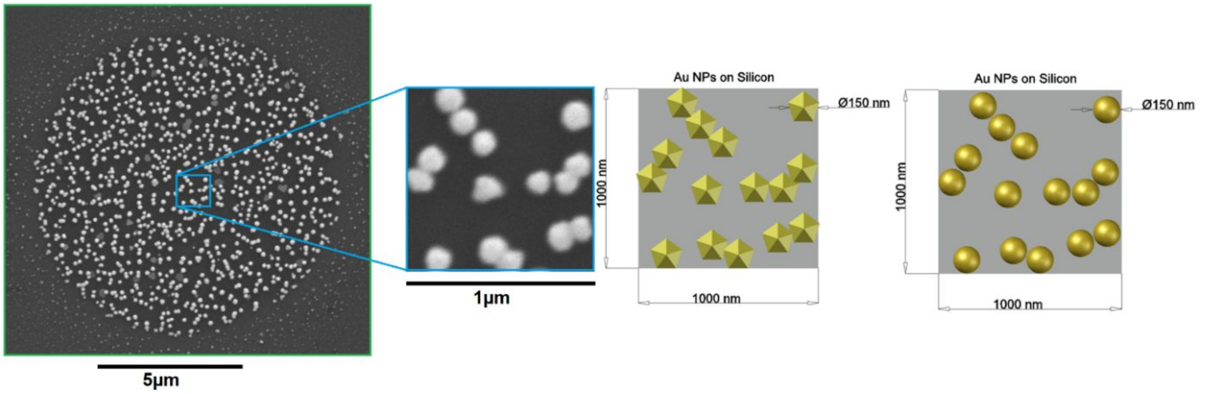

Figure S5. SEM image of NP interplays, where single nano-objects, dimers and chains are observed in the close neighbourhood. SEM is accompanied by corresponding 3D models using spherical and bipyramidal NP geometries.

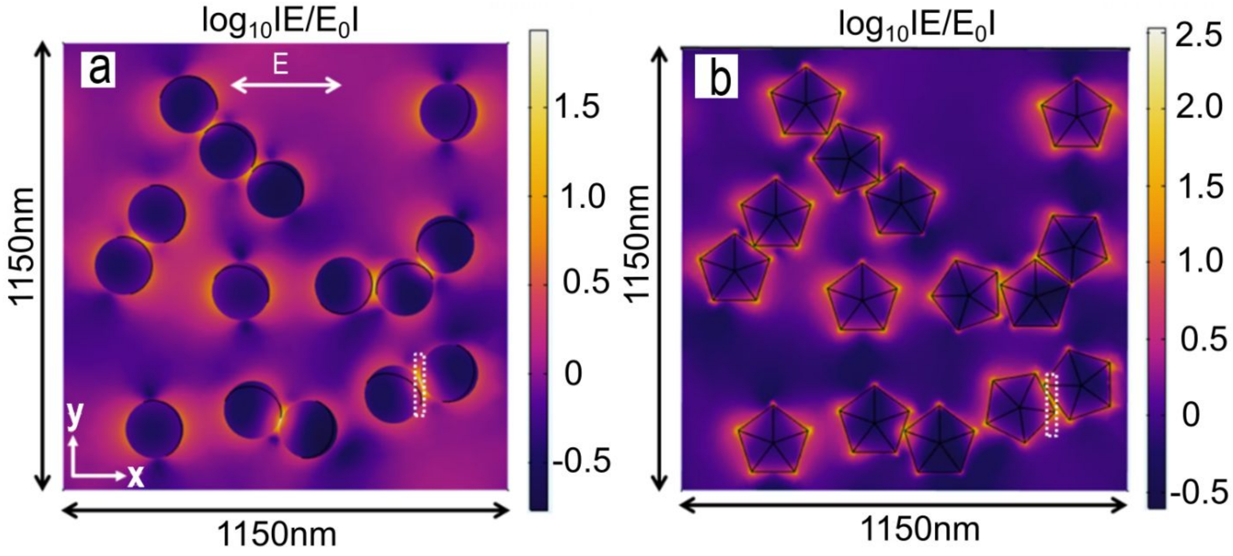

Figure S6. FD inside the unit cell of the considered periodic structure with (a) spherical and (b) non-spherical shape of the NPs. $\lambda = 633$ nm, normal incidence and TE polarisation were used to simulate the gold NPs on a silicon substrate. Air was assumed to exist between the NPs and for the superstrate. The field values are shown on a logarithmic scale.

As can be observed, the circular nano-gold pattern includes ~9 periods as the unit cell size in both *x*- and *y*-directions; this was the motivation for simulating the periodic structure. The field values in the manuscript are shown between minimum and maximum values to qualitatively show the location of the hot spots in a clear way by changing the colour bar. This provides more information regarding the calculated values of the electric field (shown in logarithmic scale) at each point in the structure as numerically achieved from the software without changing the colour bar; please see **Figure S6,** which is also important to demonstrate the fact that using the distorted NPs, higher enhancement is achieved because higher field values are observed in **Figure S6a** and **S6b.** In addition, several tests were performed to verify the accuracy and reliability of the calculation of the local FE in **Figure S6**. Average field calculations were also carried out inside defined boxes around the tiny gaps where the hot spots were observed (please see the dotted white regions in **Figure S6a** and **Figure S6b**; the box dimensions are: 30, 110, 10 nm in *x*, *y* and *z* axes, respectively). The calculations showed that a factor of ~2 enhancement in both the local and average fields in the case of the distorted NPs compared to the spheres due to the existence of the sharp edges.

The second simulation case is presented in **Figure S7** below and considers a single spherical NP cluster. **Figure S7** shows the FD inside the single spherical NP cluster for both TE (**Figure S7a**) and transverse magnetic (TM) (**Figure S7b**) polarisations at $\lambda = 633$ nm and normal incidence in which, for both cases, the largest FE (hot spots) is observed in the small gaps between the dimers and NPs forming the three NP groups. Finally, it is important to note that the FD inside the NP

cluster for the TE case (**Figure S7)**) showed similar FD behaviour as in the case of the periodic structure. This can tell us about the reliability of the simulation in regard to the FD within the NP cluster.

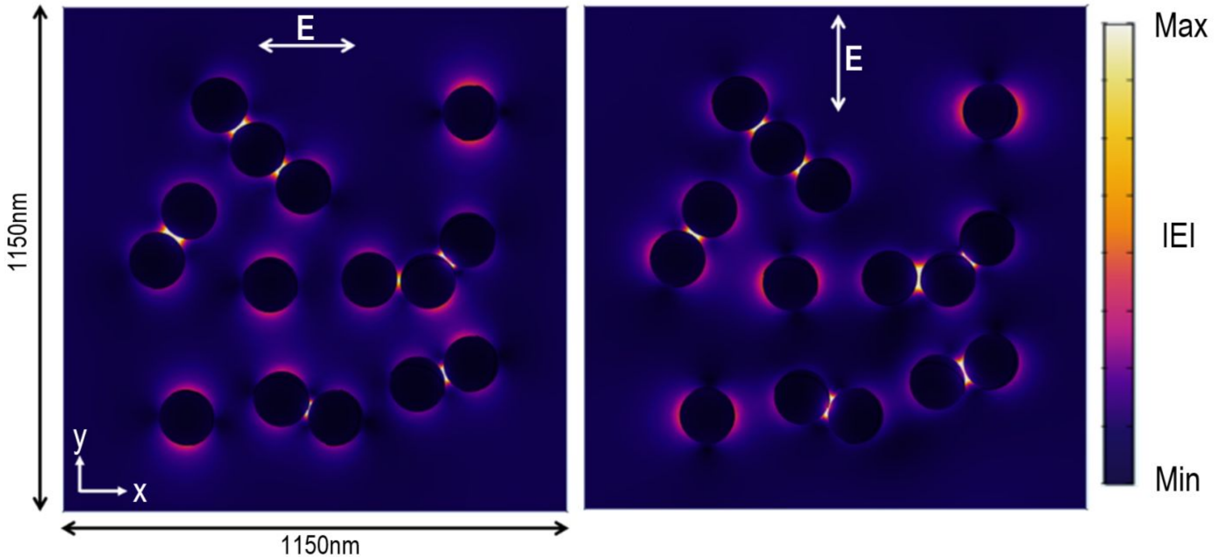

Figure S7. FD inside the single spherical NP cluster. λ = 633 nm, and normal incidence and TE polarisation (a) and TM polarisation (b) were used in the simulation of the gold NPs on a silicon substrate. Air was assumed to exist between the NPs and for the superstrate.

Bacterial DNA extraction. Genomic DNA fragments used for SERS experiments were isolated from 1.5 ml of an overnight culture of *Escherichia coli, Janthinobacterium lividum*, *Micrococcus luteus* and *Staphylococcus aureus* using GenEluteTM Bacterial Genomic DNA Kit (Merck, Darmstadt, Germany) according to the manufacturer's protocol. After the isolation procedure, DNA molecules were stored in elution solution, prepared from 10 mM Tric-HCl and 0.5 mM EDTA, pH 9.0. DNA concentration was measured using TayCell® (Hellma, Müllheim, Germany) and UV/VIS Lambda 1050 (Perkin Elmer, Massachusetts, USA). For SERS analysis, DNA solution with a concentration of 100 ± 10 ng/μl was pipetted on the silicon surface with pre-

deposited nano-gold. The genomic ratio GC% of each bacterial DNA was extracted from a data library accessible using a link (https://genomes.atcc.org/genomes).

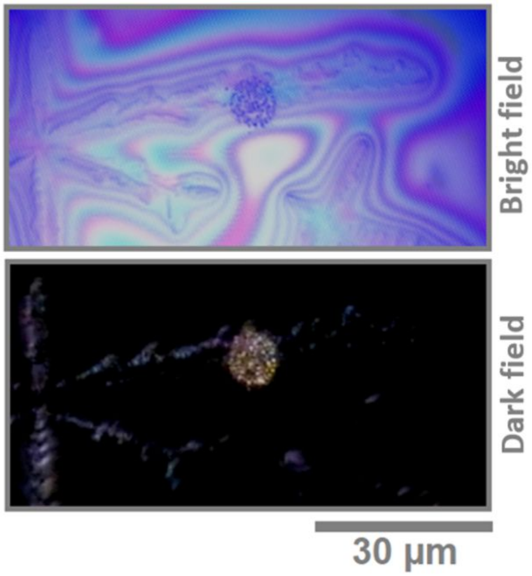

Figure S8. Optical image in bright-field and dark-field modes of the sensing substrate with dried DNA fragment deposited on top of it.

Gaussian deconvolution. Before extracting meaningful data, spectra were elaborated mathematically. Step one was a baseline subtraction using asymmetric least-squares fitting. The second procedure was to reduce noise from experimental curves by applying the locally estimated scatterplot smoothing (LOESS) function based on one main parameter: window size. Then, intensity normalisation was performed, ranging from 0 to 1 linked as minimum and maximum values. As a result, four spectra from different biospecies were obtained for Gaussian deconvolution, focusing on the most distinct Raman modes. The extructed fitting parameters are collected in **Tables S2-S4**.

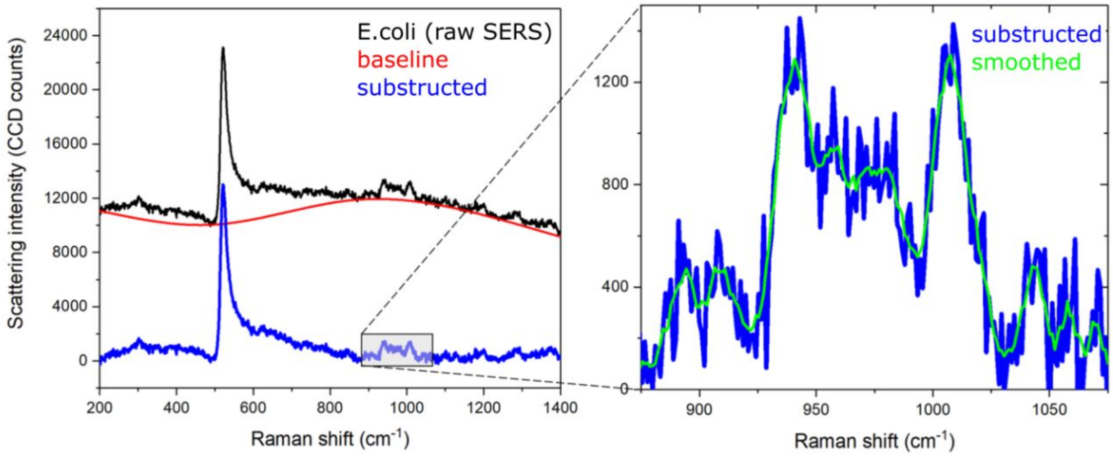

Figure S9. The preparation procedure of raw SERS spectra for PCA and Gaussian peak deconvolution.

Table S2. Relevant fitting parameters after Gaussian deconvolution for **guanine** peak.

	**S. aureus**	**E. coli**	**J. lividum**	**M. luteus**
Peak position	939.7±0.2	940.10±0.3	939.4±0.6	939.2±0.6
Peak Area	2.1±0.1	1.9±0.1	2.1±0.3	2.5±0.3
FWHM	16.7±0.6	16.3±0.4	15.9±0.6	15.8±0.7

Table S3. Relevant fitting parameters after Gaussian deconvolution for **thymine** peak.

	**S. aureus**	**E. coli**	**J. lividum**	**M. luteus**
Peak position	1008.6±0.2	1007.7±0.1	1008.1±0.2	1007.4±0.4
Peak Area	3.0±0.1	1.9±0.1	1.9±0.2	2.0±0.6
FWHM	15.9±0.3	16.2±0.5	16.1±1.2	17.0±2.8

Table S4. Relevant fitting parameters after Gaussian deconvolution for **cytosine** peak.

	S. aureus	*E. coli*	*J. lividum*	*M. luteus*
Peak position	1021.1±0.2	1021.2±0.4	1021.2±0.5	1021.2±0.1
Peak Area	0.15±0.02	0.23±0.04	0.40±0.08	0.46±0.11
FWHM	10.0±1.5	10.4±0.6	10.2±0.8	10.0±0.7

Step-by-step methodology of PC analysis. First, let's notice, that the most structurally valuable information is hidden within spectral intervals where intense peaks of guanine, cytosine and thymine occur. Thus, it was a solid reason to focus special attention on a reduced wavenumbers range of about 900-1050 cm^{-1}. Raw experimental data were elaborated similarly to a procedure employed for the spectra processing in the "Gaussian deconvolution" section. In total, 40 collected raw spectra, ten for each bacterial DNA, were baseline subtracted, smoothed, and normalised to run the principal component analysis approach. To reduce data dimensionality by multivariate ordination approach, special attention was focused on the first three principal components marked as PC1, PC2, PC3, pretending to order measured DNA spectra in a plane defined by two axes (PC1 vs PC2, PC1 vs PC3, and PC2 vs PC3) according to their extructed values. Following the well-known steps of the method (step 1: *standardisation*; step 2: *covariance matrix computation*; step 3: *compute the eigenvectors and eigenvalues of the covariance matrix to identify the principal components*), the analysis was accomplished. After the first step, needed to equalise the contribution of each continuous initial variable to the analysis, the scoring plot is obtained (**Figure S10**). Observing a graph in-depth, it can be pinpointed that PC1 asks a question, "which spectrum contains both guanine and thymine within a defined spectral range?". Subsequently, the PC2 scoring plot puts another query, "which spectrum reveals a more intense thymine Raman peak?"

Lastly, in the remaining data, PC3 is interested "which spectrum possesses a larger difference between guanine and thymine peaks?"

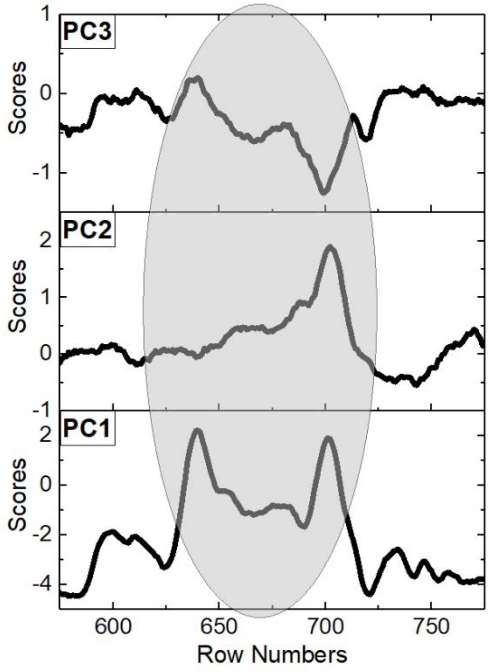

Figure S10. PCA creates lines in terms of least squares approximation by subtracting the mean and dividing by the standard deviation for each variable's value. All is reflected as PCs scores.

Afterwards of steps "2" (a covariance matrix is just a table summarising the relativeness between the created variable's pairs) and "3" (principal components are parameters constructed as linear combinations of the initial variables or their mixtures), the following graph with PCs for all spectra is obtained (**Figure S11**)

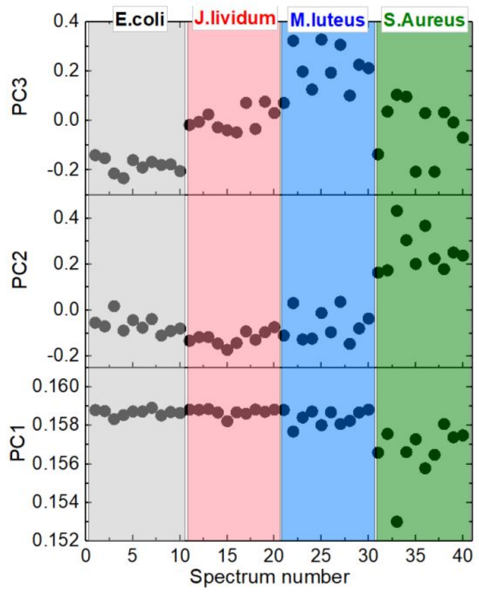

Figure S11. Calculated principal components for all spectra recorded.

It is worth noticing the values grouped better with increasing order of principal components. To visualise the clustered spectra of DNA, it is needed to take a closer look at 3D loading plots, where PCs serve as axis values (**Figure S12)**. Although the 3D plot is a bit eye-confusing, its orthogonal projection reflects the situation on quite a decent level. In particular, a combination of higher-order components PC2 and PC3 classifies all measured vibrational spectra the best regarding their fundamental genomic ratio GC%. It can be related to PCs variance between their minimum and maximum values. This difference for PC1 ($\Delta(PC1_{max}-PC1_{min}) = 0.006$) is rather small, corresponding to about 100-fold less than that found for PC2 and PC3, where $\Delta(PC2_{max}-PC2_{min}) = 0.603$ and $\Delta(PC3_{max}-PC3_{min}) = 0.562$, respectively. Consequently, a projection onto the orthogonal line using only PC1 is not sufficient to pinpoint sufficient data grouping. In contrast, orthogonal planes comprising higher principal components are way more useful for analysing a large set of datapoint (Raman spectra) interlinked by common structural features.

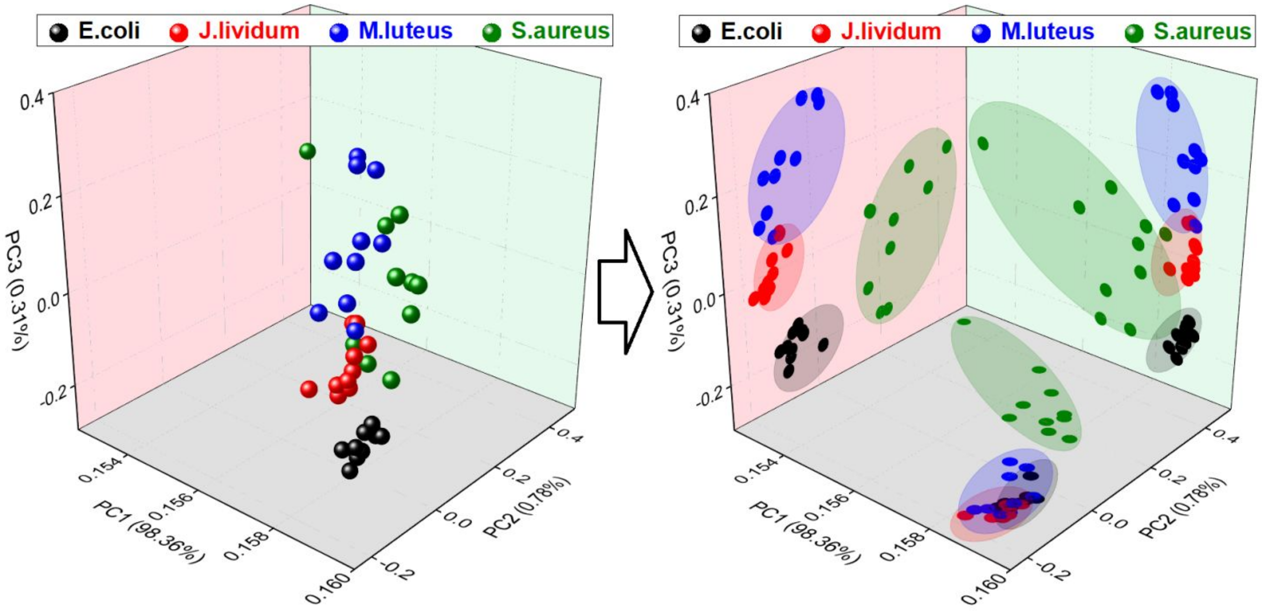

Figure S12. 3D visualisation of PCs loading plots accompanied by their projections onto main orthogonal loading planes.

DNA sequencing for GC% validation

Bacterial DNA concertation and purity were measured on NanoDrop 1000, 200 ng used for library preparation following Rapid Barcoding Sequencing protocol (SQK-RBK004, Oxford Nanopore Technologies) and sequenced on GridION sequencing device (FLO-MIN106 flowcell). Software for sequencing was MinKNOW, with Guppy basecaller. Sequencing quality was checked with NanoPlot software. Genomes were assembled with Flye software, and nucleotide content was

extracted from reads with a custom-developed algorithm written in Python. Assemblies and extracted contents were compared, where 4.73 +/- 5.52 percent difference was observed.

DNA concertation and purity were measured on NanoDrop 1000. The library was prepared according to the Rapid Barcoding Sequencing protocol (SQK-RBK004, Oxford Nanopore Technologies). 200 ng of DNA was diluted to 7,5 µl in H_2O (concentration 26,67 ng/µl), for each bacterial strain in a separated 0.2 ml thin-walled PCR tube. 2,5 µl of Fragmentation Mix was added to the sample, for fragmentation and barcoding of samples (strains and corresponding barcodes are presented in **Table S5**). Samples and Fragmentation mix were incubated for 1 min on 30˚C, 1 min on 80˚C and put on ice to cool down. Barcoded samples were then pooled in 1,5 ml Eppendorf DNA LoBind tube, 40 µl of AMPure XP (Beckman Coulter) beads were added and incubated on a Hula mixer for 5 min at room temperature. Samples were put on a magnetic stand, and the supernatant was removed, washed two times with 70% ethanol and dried on air. Beads were resuspended in 10 µl of 10 mM Tris-HCl pH 7.5 with 50 mM NaCl, 10 µl of eluate-containing library was removed from the beads on a magnetic stand. 1 µl of RAP (Rapid Adapter) was added and incubated for 5 min at room temperature. 34 µl of SQB (Sequencing Buffer), 25,5 µl of LB (Loading Beads) and 4,5 µl of H_2O was added to the library and loaded to activated flow cell. Sequencing was run on GridION sequencing device (FLO-MIN106 flowcell, Oxford Nanopore

Technologies), for 43 hours, producing a total of 1,23×10^6 fragment reads corresponding to 7.73 Gb of base pairs, with an estimated average read length 11,01 kb.

Table S5. Concentrations and sample purity with corresponding barcodes

Sample name	*S. aureus*	*M. luteus*	*J. lividum*	*E. coli*
Barcode	R1	R2	R3	R4
ng/µl	30.40	44.46	44.52	46.02
260	0.608	0.889	0.890	0.920
280	0.403	0.491	0.532	0.525
260/280	1.510	1.810	1.750	1.670
260/230	3.401	11.520	3.380	2.110
Volume for 200ng (µl)	6.58	4.50	4.49	4.35
Volume of H_2O (µl)	0.92	3.00	3.01	3.15

Raw sequencing data (*FAST5* files) was basecalled with *MinKNOW* software (version 21.11.7) on *GridION* sequencer (Oxford Nanopore Technologies). The basecaller used was *Guppy* (version 5.1.13) with a *High-accuracy basecalling* model, and sequencing reads were filtered with a

minimum quality score of 9. After basecalling, the resulting *FASTQ* files (each containing 4000 reads), were merged to form one continuous file for each of the barcoded samples.

Sequencing quality was checked with *NanoPlot* software (version 1.36.2). Quality metrics are presented in **Table S6**.

Table S6. NanoPlot experiment quality metrics

	Mean read length	Mean read quality	Read length N50	Number of reads	Number of bases
Barcode – R1	1,629.0	11.6	3,615.0	49,445	80,543,832
Barcode – R2	7,341.5	11.7	13,164.0	89,472	656,857,414
Barcode – R3	7,199.9	11.9	12,654.0	273,574	1,969,709,635
Barcode – R4	5,598.4	11.9	9,896.0	556,732	3,116,808,015
Unclassified	5,412.2	6.7	10,380.0	256,812	1,390,690,486
Experiment	5,884.5	11.0	10,881.0	1,226,168	7,215,347,868

Bacterial genomes were assembled using *Flye* assembler (version 2.8.3-b1695). Assembled genomes were circularised with exception of *R1*, where a lower number of reads with shorter

overall read length led to low genomic coverage (average contig coverage of 15x compared to more than 200x for other samples).

Assembled genomes were checked against the NCBI BLAST database (https://blast.ncbi.nlm.nih.gov/), for the best matches of the assembly to the reference genomes (**Table S7**).

Table S7: Barcode to NCBI BLAST database comparison

Barcode	Reference name	Identification percentage
R1	Staphylococcus aureus	99.97%
R2	Micrococcus luteus	99.98%
R3	Janthinobacterium lividum	97.30%
R4	Escherichia coli	99.99%

An algorithm for calculating separate nucleotide content was written and developed in Python programming language (version 3.7.3). The calculation was done in reads through time. Reads were grouped by time passed (every minute), and nucleotide content was calculated for each group.

The output of the algorithm presented how nucleotide content was changing through time (**Figure S13**).

The nucleotide content of reads was further compared to the nucleotide content of assembled genomes. The observed differences (4.73 +/- 5.52) can be attributed to the assembly method/algorithm and other plasmids/genomes in the sequencing library. In contrast, when assembled genomes were compared to the database references (https://www.patricbrc.org), differences in GC content were 0.11 +/- 0.098 percent.

The outlier in the last comparison was barcode *R1.* There the assembly fragments (although not circularised) GC nucleotide content was similar to the whole genome sequencing reference, but read GC content differed by a higher margin (5.35 percent). The cause of this could be attributed to sampling quality or contamination.

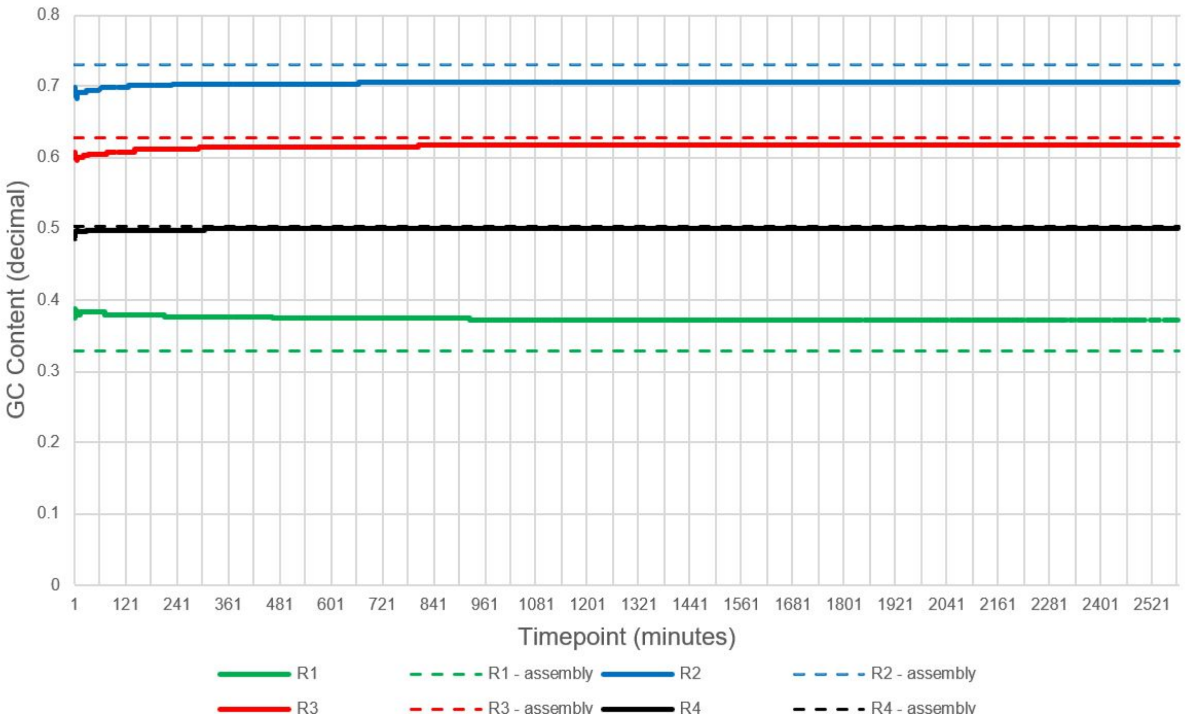

Figure S13. GC nucleotide content of reads and assembled genomes. Assembled genome GC nucleotide content (dashed line) does not change because it represents total genomic GC content.
